# The calcium cyanamide and polyethylene blocks the secondary transmission and infection of vegetable leaf diseases

**DOI:** 10.3389/fpls.2022.1027584

**Published:** 2022-12-20

**Authors:** Xuewen Xie, Lida Chen, Yanxia Shi, Ali Chai, Tengfei Fan, Lei Li, Baoju Li

**Affiliations:** ^1^Institute of Vegetables and Flowers, Chinese Academy of Agricultural Sciences, Beijing, China; ^2^College of Horticulture, Gansu Agricultural University, Lanzhou, China

**Keywords:** *Corynespora cassiicola*, vegetable residues, propidium monoazide (PMA), calcium cyanamide, polyethylene, transmission

## Abstract

Continuous cropping obstacles, especially soil-borne diseases can cause serious harm to agricultural production and limit the sustainable development of modern agriculture. However, Corynespora blight is an important air-borne disease on cucumber leaves caused by *Corynespora cassiicola.* The pathogen also could survive in air-dried soil or plant residue for at least one month. However, it is not clear whether soil Corynespora blight residues can infect plants. We detected the dynamic change of C. cassiicola content in soil and air after returning the diseased and residual straw to the field in real time by PMA-qPCR detection method. In this study, we reveal for the first time a new mode of transmission in which leaf blade disease residues in soil can spread again into the air and infect plants. In polyethylene (PE) treatment, cucumber plants grew healthily without disease. However, the content of *C. cassiicola* in the soil still existed in the PE treatment at 10^3^ spore·g^−1^. The disease index (DI) of cucumber was less than 3 in calcium cyanamide (CaCN_2_). After fumigation and film removal and the whole growth period was controlled at a safe level. In addition, the PMA-qPCR detection method of Corynespora blight of cucumber was established for the first time in this study. In summary, CaCN_2_ and PE treatments are effective ways to block the infection of cucumber leaves by Corynespora blight residues in soil. These treatments are considered to comprise a feasible and sustainable technique for vegetable leaf residues in greenhouses.

## 1 Introduction

Cucumber is an important vegetable crop in China, and its cultivation area accounts for approximately 60% of the world’s total cucumber cultivation area (FAOSTAT; http://www.fao.org/faostat/en/#home). Greenhouse planting is the main mode of cucumber production in China. Solar greenhouses and plastic greenhouses are the most popular cultivation facilities in China, particularly in northern China. In these greenhouses, cucumber plants are often cropped continuously and are planted once or twice a year (Chai et al., 2020b). This large-scale cultivation produces considerable amounts of vegetable residue, which is rich in nutrients and organic matter. Five main utilization approaches for residue have been proposed by the Ministry of Agriculture, i.e., use as fuel, fertilizer, feed, industrial raw material, and base material. Returning residue into soil can improve soil fertility, reduce chemical fertilizer input (Li et al., 2018; Wei et al., 2021) and directly reduce environmental pollution caused by the illegal activities of residue burning (Jiang et al., 2021). In agricultural production, it is customary to remove the straw of greenhouse crops from the shed. However, falling diseased leaves in the soil are often ignored, leads to high water content and the possibility of carrying detrimental pathogens (Fernández-Gómez et al., 2010). It can provide a suitable environment for the growth, reproduction and accumulation of pathogens, leading to plant diseases (Govaerts et al., 2007; Yuan et al., 2021). Pathogens can survive as resting hyphae in the soil or diseased bodies for at least five years (Chen et al., 2022). Under favorable conditions, the resting hyphae are activated, after which they colonize tissues and kill the young seedlings before or soon after emergence. At present, research on plant infection by soil disease residues mainly focuses on soil-borne diseases. Can leaf diseases become soil disease residues that infect plant leaves again?

Corynespora blight, caused by *Corynespora cassiicola*, was first reported in European countries in 1906 (Gussow, 1906) and has become one of the most important plant leaf diseases affecting cucumber production (Liu et al., 2019). *C. cassiicola* can infect more than 530 plant species, including cucumber, tomato, and cowpea, resulting in huge economic losses in more than 70 countries (Dixon et al., 2009). *C. cassiicola* mainly infects healthy leaves through air transmission and seed carriers (Lee et al., 1990a; Goulart and Utiamada, 2020). The pathogen could remain alive on plant residue into soil about several months, which caused the pathogen aggravated year after year (Erick and Scott, 2007; Mi et al., 2010). Although plant seedlings, air and diseased plants are disinfected and sterilized, Corynespora blight diseases still occur after transplanting seedlings every year. Therefore, it is preliminarily suspected that the Corynespora blight disease originates from the soil residues. At present, there is no direct evidence to prove that the transmission of aboveground foliar diseases is caused by the return of leaf residues to the soil.

Recently, the main methods for using vegetable residues have been anaerobic digestion (Ros et al., 2017) and composting (Cui et al., 2020). Due to the complexity of the anaerobic digestion process and its equipment requirements, this process has not been widely applied in the process of vegetable residue use (Bouallagui et al., 2009). Moreover, the disadvantages of composting include transportation, site availability, labor requirement, and bad smell (Wang et al., 2020). Hence, the exploration of feasible and economic techniques for the use of vegetable residue is urgently needed.

Calcium cyanamide (CaCN_2_) is not only a good fertilizer but also a good suppressor of soil-borne pathogens (Shi et al., 2009). CaCN_2_ breaks down into hydrogen cyanamide in the soil, which is highly toxic to soil microbes (Shi et al., 2009). Application of CaCN_2_ effectively suppresses clubroot disease in cabbage, fusarium wilt in cucumber and melon, etc. (Shi et al., 2009). Covering plants with polyethylene film is common in agricultural production; while this action can prevent the transmission of disease residues in the soil by air, it can also cause plant diseases and play a role in isolation (Yoshiaki, 1994). Whether the above two soil treatments have a good control effect on soil residues remains worth studying.

Therefore, we determined the basis for whether the disease residues in the soil spread to the air and infect cucumber leaves. We aimed to explore the inhibitory effect of lime nitrogen and plastic film treatment technology on the transmission of soil disease residues. In addition, we established a PMA-qPCR detection method for *C. cassiicola* to analyze the content of *C. cassiicola* in soil and air for the first time. Our results will provide a better understanding of the technical feasibility of the application of vegetable residue return in the Chinese solar greenhouse.

## 2 Materials and methods

### 2.1 Establishment and application of PMA-qPCR for the detection of *C. cassiicola*

#### 2.1.1 Pathogen strains, primer design and qPCR conditions

In total, 8 strains, including 1 C*. cassiicola* strain and 7 nontarget strains, were used for primer development and specific detection (**Supplementary Table S1**). Cultures were grown in potato dextrose agar (PDA) medium at 26°C for 5 days. Genomic DNA was extracted by the CTAB method (Lee and Taylor, 1990b). Conidia were collected with a sterile solution of Tween 80 (0.005% v/v) and filtered through Miracloth (Calbiochem, USA). The conidial concentration was determined using a Thoma counting chamber and then stored at 4°C.

Primers CC-F3 (5′-CAGGAAATCCTCGCCAAGCAG-3′) and CC-R3 (5′-CGCCAGTGATACGGTTGAACGG-3′) were designed for specific amplification of a segment 109 bp in length of *C. cassiicola* DNA based on alignments of *C. cassiicola* SdhB sequences (GenBank accession numbers MG729615.1).

The 20 μL reaction volume of PCR contained template DNA (1 μL), Premix Taq™ (10 μL) and 10 μmol·L^−1^ primer (0.4 μL of each). Initial denaturation was performed at 94 °C for 5 min, followed by 35 cycles of denaturation at 94 °C for 30 s, annealing at 58 °C for 30 s, and extension at 72 °C for 45 s with a final extension of 10 min. A 20 μL reaction volume of qPCR containing template DNA (1 μL), SuperReal PreMix Plus (TIANGEN Biotech) (10 μL), 10 μmol·L^−1^ primer (each) and 50×ROX Reference Dye (0.4 μL) was used. The amplification procedure was consistent with the method described by Chen et al. (2022).

#### 2.1.2 Establishment of a standard curve and sensitivity test

*C. cassiicola* was inoculated into PDA medium, and the culture was activated at 28.5°C for 5 d. The spores on the plate were washed with sterile water to prepare spore suspensions with concentrations of 10^7^ spores·mL^-1^. Standard curves were constructed, and the sensitivity was determined according to the method described by Chen et al. (2022).

#### 2.1.3 Optimization of PMA concentration and light duration

The PMA concentration and exposure time were optimized according to the method described by Chen et al. (2022). A shaker was used to mix thoroughly to obtain a mixture of PMA, dead and viable cells with final concentrations of 0, 10, 20, 40, 80 and 100 μmol·L^−1^. The exposure time of the halogen lamp was set to 0, 2, 5, 10 and 20 min for 5 treatments. The PMA-qPCR detection of each sample was repeated 3 times to ensure the repeatability and reliability of the test.

#### 2.1.4 Detection and application of PMA-qPCR in suspension and soil

A suspension of 10^7^ spores·mL^−1^ was completely mixed to prepare 0%, 25%, 50%, 75% and 100% viable cell suspensions. The mixed suspension (200 μL each) was detected by qPCR and PMA-qPCR methods.

The soil was sterilized at 121°C for 20 min, and then suspensions of *C. cassiicola* spores were treated in distilled water containing Tween 80 (0.005% v/v). The soil (200 g) was infected with 200 mL of spore suspensions (10^7^, 10^6^, 10^5^, 10^4^, 10^3^, 10^2^ and 10 spore/g soil) and homogenized for 60 s. Another 200 g of sterilized soil not inoculated with *C. cassiicola* and infected soil with 10^7^ dead spores was used as the negative control. Infection of the soil by *C. cassiicola* was analyzed with qPCR and PMA-qPCR methods.

### 2.2 Transmission and blocking experiment of plant debris in greenhouses

The four treatments included the following. (1) CC: plant debris was mixed with sterile soil at a ratio of 1:4 by weight and placed in pots (Barak and Liang, 2008). (2) CC-PE: plant debris was manually mixed into the soil and covered with polyethylene film (PE film, 0.04-μm thick, Shandong Longxing Science and Technology Co. Ltd., China) for the duration of the test. The treated soil was sprayed gently with water to reach 60% relative humidity (RH). (3) CC-CaCN_2_: plant debris was manually mixed into the soil, and CaCN_2_ (120 g/m^2^) was manually injected into the soil and covered with PE film for 15 d. (4) CK: natural soil without any treatment. Each treatment contained three replicates, and 16 cucumber seedlings were planted in each replicate. Corynespora blight samples were collected from the experimental field in Shouguang city, Shandong Province. Plant leaf debris naturally infected by *C. cassiicola* was collected from a field and cut into pieces 0.5 cm long. The debris was mixed with the soil and placed into a pot (*φ*=10 cm, height 10 cm). In the CaCN_2_ treatment, the soil was sterilized with Roebon^®^ (CaCN_2_), Ningxia Darong Industrial Group Co., Ltd, China). The soil was placed into pots and CaCN_2_ was evenly spread on the soil surface, with high RH (60%), at a rate of 120 g/m^2^. Then, the soil was mixed thoroughly and immediately covered with PE film to maintain a high soil temperature and humidity. After 15 d of soil fumigation, the PE plastic film in the CaCN_2_ treatment was removed and dried for 5 d to transplant cucumber seedlings.

#### 2.2.1 Cucumber planting

Cucumber seeds (‘Cucumber Zhongnong No. 6’, produced by the Institute of Vegetables and Flowers, Beijing, China) were initially sown in seedling plug trays. Two weeks after seedling growth, the seedlings were removed from the trays and planted in each pot. The seedlings of different treatments were cultured in an aerosol chamber made of organic glass with a size of 70 cm × 60 cm × 60 cm (length × width × height). The specifications of the aerosol chamber are described by Chai et al. (2020a). In briefly, the Andersen sampler was connected to the air outlet located at the sidewall to collect bioaerosol samples. A door (20 cm × 20 cm) was fixed at the front wall with six screws, and a silicone pad was applied to ensure the chamber was airtight. The air temperature was controlled by an air conditioner (Haier, KFRd-27N/PAA12, China). The humidity was controlled by an air humidifier (Yadu, SC-EB35B, China) through a channel preinstalled with a HEPA filter. A UV light was installed in the chamber for sterilization before each experiment. In order to avoid pathogen coming from the environment, 75% alcohol is used to disinfect the greenhouse air before the test treatment, and the air is naturally dried for 1 day. In addition, the soil used was sterilized by pressure cooker at 121 °C and then the vegetable residue into soil. The healthy cucumber seeds, soil, aerosol chamber and greenhouse air used in this study were first confirmed to be free of *C. cassiicola* by traditional agar planting method and PMA-qPCR.

#### 2.2.2 Determination of soil and air pathogens

Soil samples were taken five times during the cucumber season: 0 days after treatment (DAT), 15 DAT, 25 DAT, 30 DAT, and 35 DAT. Five samples were randomly taken from each soil treatment as a composite sample, and the sampling of each treatment was repeated three times. Soil samples from 0–10 cm deep were randomly collected from each treatment, mixed evenly and sent to the laboratory. The concentrations of *C. cassiicola* in soil and air samples in the aerosol chamber were determined by PMA-qPCR. In order to avoid spores in indoor environment, the collection time and detection method of ambient air are consistent with those of soil. The air aerosol-containing *C. cassiicola* collection method was described by Chai et al. (2020a). Briefly, air aerosols were collected onto φ=90 mm sterilized aluminum membrane-coated (coated 600 μL of mineral oil). Then, the samples were immediately transferred to a 50-mL centrifuge tube, washed using 3 mL of Tween 20 suspension and pretreated with PMA. Genomic DNA was extracted from the obtained suspension using a TIANamp fungal DNA Kit (Tiangen Biotech, Inc., Beijing, China) according to the manufacturer’s guidelines, and then the bioaerosol was analyzed by PMA-qPCR.

### 2.3 Transmission and blocking experiment of plant debris in the field

The research was performed in a greenhouse in 2021 in Shouguang County, Shandong Province, China (N 37°11′ and E 118°48′). Cucumber debris naturally infected by *C. cassiicola* was collected from a field and cut into pieces 3 cm long. The twelve plastic arch shed were set out as a randomized complete block with three replicates and four treatments: (1) CC: the returning density of cucumber debris was 4 plants·m^-2^) (Wei et al., 2021). (2) CC-PE: plant debris was manually mixed into the soil and covered with PE film for the duration of the test. The treated soil was sprayed gently with water to reach 60% relative humidity (RH). (3) CC-CaCN_2_: CaCN_2_ (120 g/m^2^) and plant debris were artificially mixed 15–20 cm into the soil, and then a small rotary tiller was used to evenly mix the soil. The samples were covered with PE film, and a soil humidity of 60% was maintained during fumigation. The film was pressed into the soil around the edges to minimize gas loss. The film was lifted after 15 d, and cucumber was planted after 5 d of natural drying. (4) CK: healthy soil without returning residue. During the test, fluopyram fungicide was sprayed regularly to disinfect greenhouse corridors and air spray. The healthy cucumber seeds, soil and air in plastic arch shed used in this study were confirmed to be free of *C. cassiicola* by traditional agar planting method and PMA-qPCR.

#### 2.3.1 Cucumber planting

The experiment was a completely randomized block design with three replicates, and the size of each replicate plot was 9 m×3 m. Cucumber was transplanted by hand in each treatment (four rows, 35 cm between rows, 30 cm between plants). Furrow irrigation systems were adopted in the greenhouse based on the conventional schedule. No fertilizer was applied during the summer cover crop planting season. Ninety-six cucumber seedlings were planted in each plot. Field management followed conventional practices. Weeds were removed by hand. The bupirimate and dimethomorph fungicide sprays were used to control powdery mildew and downy mildew respectively, which frequently occur during cucumber growth.

#### 2.3.2 Determination of soil and air pathogens

The soils were sampled after 0, 1, 5, 15, 20, 35, 50, 65 and 70 DAT during the cucumber season. Five samples were randomly taken from the soil 0–10 cm deep in each treatment as a composite sample, and each treatment was repeated three times. The concentrations of *C. cassiicola* in soil and air samples in the plastic arch shed were determined by PMA-qPCR, respectively.

### 2.4 Disease investigation

Disease development was evaluated starting from the first appearance of symptoms. The percentage of leaf area covered by lesions was estimated visually, and disease severity was assessed following the severity assessment scale described by Shaner and Finney (1977), where 0 = 0%; 1 = 1–5%; 2 = 6–25%; 3 = 26–50%; and 4 = >50% leaf area covered by lesion.

### 2.5 Statistical analysis

The differences among each group were compared using one-way ANOVA, followed by Tukey’s *post-hoc* test. P values less than 0.05 were considered statistically significant. The level of significance (α) was set to 0.05 for all tests.

## 3 Results

### 3.1 Establishment and application of the PMA-qPCR detection system

#### 3.1.1 Establishment of the PMA-qPCR detection system

**Figure 1:**
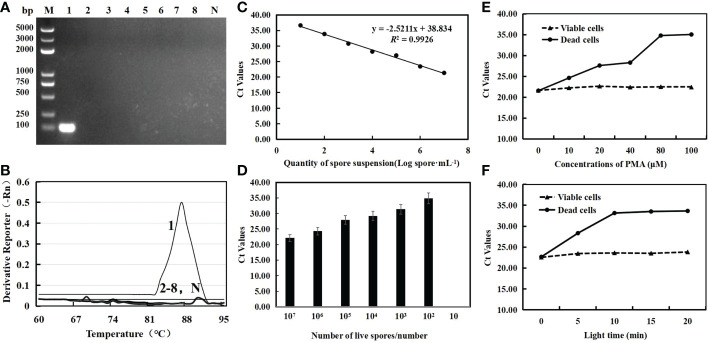
Specific detection for primer CC-F3/CC-R3 where M is a ladder (100 bp) DNA ladder **(A)** and the melting curve of qPCR for gradient dilution genome DNA of *C*. *cassiicola* **(B)**; lane 1 is *C*. *cassiicola* samples, lane 2 is *Pseudoperonospora cubensis* samples, lane 3 is *Sphaerotheca cucurbitae* samples, lane 4 is *Pseudomonas syringae* samples, lane 5 is *Fusarium oxysporum* samples, lane 6 is *Alternaria cucumerina* samples, lane 7 is *Cladosporium cucumerinum* samples, lane 8 is *Colletotrichum orbiculare* samples, and lane N is ddH_2_O. PMA-qPCR standard curve **(C)** and sensitivity analysis **(D)**; Effects of PMA concentrations **(E)** and light time **(F)** on the amplification of DNA from viable and dead *C. cassiicola* spores at 10^7^ spore·mL^−1^. Ct, cycle threshold.

Only the 109 bp specific band was amplified from *C. cassiicola*, while other cucumber disease pathogens showed no bands (**Figure 1A**). In addition, the qPCR test found that *C. cassiicola* had a unique amplification curve (Ct=18.37), while other strains and negative controls had no amplification curve (**Figure 1B**). The standard curve (y =-2.5211x+38.834) was drawn with the spore value per mL as the abscissa and the Ct value as the ordinate. The correlation coefficient was 0.9926 (**Figure 1C**). The lower limit of the sensitivity of qPCR detection was 100 spores/mL (**Figure 1D**). This shows that the specific primers CC-F3/CC-R3 had good specificity and sensitivity.

The concentration and light time had no significant difference in the Ct values of viable *C. cassiicola* cells. In contrast, when the PMA concentration was less than 80 μmol·L^-1^, the Ct value of PCR amplification of dead fungi increased with increasing PMA concentration. When the PMA concentration was ≥80 μmol·L^−1^, the Ct value did not change significantly (**Figure 1E**). When the exposure time was extended to 10 min, the inhibitory effect on dead fungal DNA increased, and the Ct value increased significantly, but there was no significant difference in the Ct value of each treatment with an exposure time of more than 10 min (**Figure 1F**). Thus, 80 μmol·L^−1^ PMA at 10 min of light time would be the most suitable final concentration, which can completely inhibit the amplification of DNA from dead cells.

#### 3.1.2 Application of PMA-qPCR in suspension and soil

There was no significant change in different concentrations of cells by qPCR. This result indicated that the qPCR detection method cannot distinguish between dead and viable cells. In contrast, the PMA-qPCR method showed significant differences in the detection of different proportions of suspension. When the ratio of viable cells decreased from 100% to 25%, the Ct values significantly increased from 21.34 ± 0.40 to 25.98 ± 0.64, and the corresponding viable cell counts decreased significantly from 6.94 ± 0.16 to 5.10 ± 0.25 log spore·mL^−1^ (*p* < 0.05). When the ratio of viable cells was 0%, the DNA was not amplified (Ct=0) by PMA-qPCR. The results show that for the mixture of dead fungi and live fungi, the detection of PMA-qPCR can completely inhibit the amplification of dead fungal DNA in the mixture but has no effect on the amplification of live fungal DNA (**Table 1**).

**Table 1:** Comparison of total and viable *C. cassiicola* spore counts obtained by different methods ^a^.

Ratio ^b^	qPCR		PMA-qPCR	
	Ct values	Total spore count	Ct Values	Viable spore count
100%	19.58 ± 0.48	7.64 ± 0.19 a	21.34 ± 0.40	6.94 ± 0.16 a
75%	20.43 ± 0.39	7.30 ± 0.16 a	22.47 ± 0.71	6.49 ± 0.28 b
50%	20.20 ± 0.81	7.39 ± 0.32 a	23.05 ± 0.31	6.26 ± 0.12 b
25%	20.44 ± 0.49	7.29 ± 0.19 a	25.98 ± 0.64	5.10 ± 0.25 c
0%	20.94 ± 0.62	7.10 ± 0.24 a	0	0 d

^a^ Values represent the means ± standard deviations of three replicates. The total spore concentration (including viable and dead cells) was 1 × 10^7^ spores/mL. qPCR = quantitative real-time PCR, PMA = propidium monoazide, and Ct = cycle threshold. Total and viable spore counts are shown as log spores per milliliter. Columns labeled with different letters indicate statistically significant differences (p < 0.05).

^b^ Ratio of viable spores. The same applies below.

The highest number of viable *C. cassiicola* spores was detected in soil samples contaminated with a fungal suspension of 10^7^ spores·mL^−1^. The content of *C. cassiicola* in the soil was detected by the PMA-qPCR method and was 3.76×10^6^ spores·g^−1^. With the decrease in the number of viable cells in soil infected with *C. cassiicola*, the detection values of the PMA-qPCR method decreased gradually. The soil infection was minimal with a *C. cassiicola* suspension of 10^2^ spore·mL^−1^, with approximately 96.44 spore·g^−1^ of soil by PMA-qPCR. However, the content of *C. cassiicola* in the soil was not detected by the PMA-qPCR method, which included 10 spores·g^−1^, 10^7^ dead spores·g^−1^ and CK treatment (**Table 2**). Therefore, PMA-qPCR was more sensitive in the detection of viable soil *C. cassiicola*.

**Table 2:** Determination of viable cells in soil samples artificially infected with *C. cassiicola* by different methods ^a^.

Infected (spore·mL^-1^)^b^	PMA-qPCR	
	Ct values	Viable spore counts
1×10^7^ viable spores	22.34 ± 0.44 a	3.76×10^6^
1×10^6^ viable spores	24.40 ± 0.22 b	5.44×10^5^
1×10^5^ viable spores	28.59 ± 0.50 c	1.30×10^4^
1×10^4^ viable spores	30.18 ± 0.80 d	3.57×10^3^
1×10^3^ viable spores	33.81 ± 0.55 e	1.09×10^2^
1×10^2^ viable spores	34.13 ± 0.93 e	96.44
10 viable spores	>35 f	Not available
10^7^ dead spores	>35 f	Not available
0 (CK)	>35 f	Not available

^a^ Viable spore counts are shown as spores per gram of soil. Not available means the C. cassiicola spore counts were zero or below the detection limit.

^b^ Artificially infected soil.

### 3.2 Soil treatment blocked the transmission and infection of greenhouse cucumber leaf diseases

The PMA-qPCR method was used to detect the content of *C. cassiicola* in different soil treatments. The content of *C. cassiicola* in the CC treatment displayed a decreasing trend during the test, which decreased from 7.10×10^7^ spore·g^−1^ at 0 DAT to 1.02×10^5^ spore·g^−1^ at 35 DAT. The same trend was observed in the soil from the CC-PE treatment, which decreased from 7.10×10^7^ spore·g^−1^ at 0 DAT to 1.10×10^3^ spore·g^−1^ at 35 DAT. No *C. cassiicola* content was detected after 15 DAT in the CC-CaCN_2_ treatment, and 151 spores·g^−1^ of soil *C. cassiicola* were detected until 35 DAT. *C. cassiicola* was not detected in the CK treatment during the test (**Table 3**). These results indicate that CC-CaCN_2_ and CC-PE, especially CC-CaCN_2_, more effectively inhibited the growth of soil *C. cassiicola.*

**Table 3:** The *C. cassiicola* content of soil and air was detected in different treatments (spore·g^−1^).

Treatment ^a^	Sample	0 DAT ^b^	15 DAT	25 DAT	30 DAT	35 DAT	DI ^c^
CC	Soil (spore·g^−1^)	7.10×10^7^	3.72×10^6^	1.36×10^6^	2.24×10^5^	1.02×10^5^	48.27
	Air (spore·m^3^)	0	224	2623	2753	3366	
CC-PE	Soil (spore·g^−1^)	7.10×10^7^	7.17×10^4^	1.06×10^4^	2.51×10^3^	1.10×10^3^	0
	Air (spore·m^3^)	0	0	0	0	0	
CC-CaCN_2_	Soil (spore·g^−1^)	7.10×10^7^	0	0	0	151	0
	Air (spore·m^3^)	0	0	0	0	0	
CK	Soil (spore·g^−1^)	0	0	0	0	0	0
	Air (spore·m^3^)	0	0	0	0	0	
Indoor environment (spore·m^3^)		0	0	0	0	0	–

^a^ Treatment: CC: plant debris was mixed with sterile soil. CC-PE: plant debris was mixed into the soil and covered with polyethylene film. CC-CaCN_2_: plant debris was mixed into the soil, and CaCN_2_ was added. CK: natural soil without any treatment.

^b^ DAT, day after treatment.

^c^ DI, disease index. -,denotes no practical significance.

The PMA-qPCR method was used to detect the presence of *C. cassiicola* in the air of different soil treatments. The *C. cassiicola* content of air in the CC treatment displayed a sharp increasing trend during the test, increasing from 224 spore·m^3^ at 0 DAT to 3366 spore·m^3^ at 35 DAT. However, the content of *C. cassiicola* in the air was not detected in the CC-PE, CC-CaCN_2_ and CK treatments (**Table 3**). In addition, no *C. cassiicola* spore content was detected in the indoor environment during the entire test period. These results indicate that the CC-CaCN_2_ and CC-PE treatments could block the transmission of *C. cassiicola* in the soil to the air.

**Figure 2:**
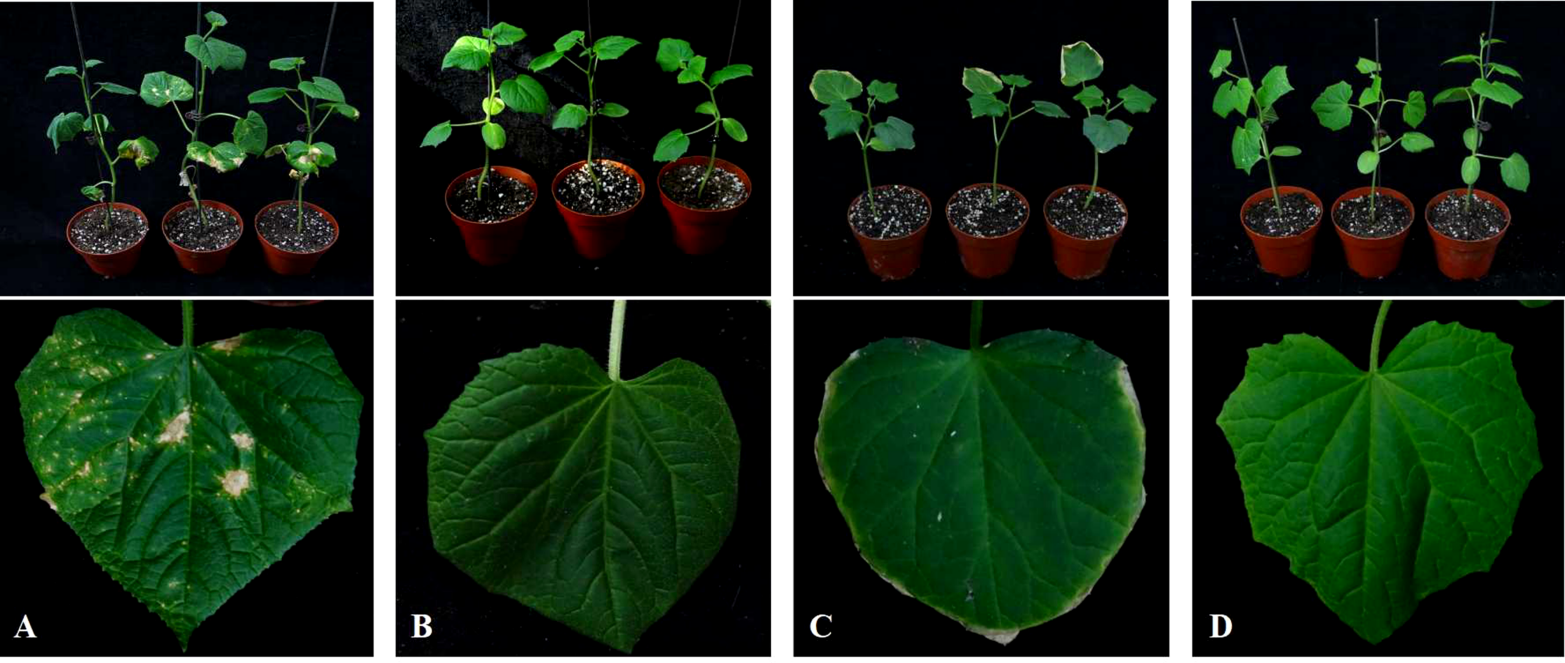
Effects of different soil treatments on leaf spot of cucumber seedlings. **(A)** Plant debris was mixed with sterile soil (CC). **(B)** Plant debris was mixed into the soil and covered with polyethylene film (CC-PE). **(C)** Plant debris was mixed into the soil, and CaCN_2_(CC-CaCN_2_) was added. **(D)** Natural soil without any treatment (CK).

The DI of cucumber plants in different soil treatments was investigated at 35 DAT. The DI of cucumber Corynespora blight, which showed symptoms of severe yellow leaf spots in the CC treatment, was 48.27 (**Figure 2**; **Table 3**). The DI of the other treatments was 0, indicating that CC-PE and CC-CaCN_2_ treatments could prevent *C. cassiicola* in soil from spreading through the air and infecting cucumber leaves. Notably, cucumber leaves show slight phytotoxicity in the CC-CaCN_2_ treatment (**Figure 2**).

### 3.3 Soil treatment blocked the transmission and infection of field cucumber leaf diseases

The content of *C. cassiicola* in different soil treatments was determined by PMA-qPCR after returning the plant debris to the field. During the whole test, the content of soil *C. cassiicola* in the CC treatment was at a high level of 10^5^ – 10^7^ spores·g^−1^. CC-PE treatment was still at a high level of 10^4^ spore·g^−1^ from 0 DAT to 20 DAT. However, the content of *C. cassiicola* in the CC-PE treatment displayed a decreasing trend after 20 DAT, which decreased from 2.11×10^5^ spore·g^−1^ at 20 DAT to 1.68×10^3^ spore·g^−1^ at 70 DAT. The content of soil *C. cassiicola* in the CC-CaCN_2_ treatment decreased rapidly at 0 – 5 DAT and was not detected at 15 and 20 DAT. However, it recovered slowly from 20 – 55 DAT to 10^2^ – 10^3^ spore·g^−1^. *C. cassiicola* was not detected in the CK treatment during the test (**Table 4**).

**Table 4:** The *C. cassiicola* content of soil and air was detected in different treatments.

Treatment ^a^	Sample	0 DAT ^b^	1 DAT	5 DAT	15 DAT	20 DAT	35 DAT	50 DAT	65 DAT	70 DAT
CC	Soil (spore·g^−1^)	2.03×10^7^	9.10×10^6^	2.63×10^6^	3.10×10^6^	7.72×10^6^	2.59×10^5^	2.12×10^5^	2.58×10^6^	4.51×10^5^
	Air (spore·m^3^)	0	0	1.60×10^3^	2.83×10^3^	8.60×10^3^	3.30×10^3^	6.73×10^3^	9.80×10^5^	4.13×10^4^
CC-PE	Soil (spore·g^−1^)	2.00×10^7^	1.12×10^6^	1.28×10^6^	5.93×10^4^	2.11×10^5^	1.44×10^4^	2.26×10^4^	5.60×10^3^	1.68×10^3^
	Air (spore·m^3^)	0	0	0	0	0	0	0	0	0
CC-CaCN_2_	Soil (spore·g^−1^)	1.79×10^7^	2.29×10^5^	2.27×10^4^	0	0	2.69×10^3^	1.88×10^3^	4.83×10^2^	1.58×10^3^
	Air (spore·m^3^)	0	0	0	0	0	0	1.02×10^3^	4.90×10^2^	5.07×10^2^
CK	Soil (spore·g^−1^)	0	0	0	0	0	0	0	0	0
	Air (spore·m^3^)	0	0	0	0	0	0	0	0	0

^a^ Treatment: CC: plant debris was mixed with sterile soil. CC-PE: plant debris was mixed into the soil and covered with polyethylene film. CC-CaCN_2_: plant debris was mixed into the soil, and CaCN_2_ was added. CK: natural soil without any treatment.

^b^ DAT, day after treatment.

The content of airborne *C. cassiicola* detected in the CC treatment was approximately 10^3^ spore·m^3^ at 5 – 50 DAT. However, the content gradually increased at 65 DAT and 70 DAT to 9.80×10^5^ spore·m^3^ and 4.13×10^4^ spore·m^3,^ respectively. In the CC-CaCN_2_ treatment, no *C. cassiicola* was detected at 0 – 35 DAT until it was detected at 50 – 70 DAT, which was 10^2^ – 10^3^ spore·m^3^. *C. cassiicola* was not detected in the CC-PE and CK treatments during the test (**Table 4**).

**Figure 3:**
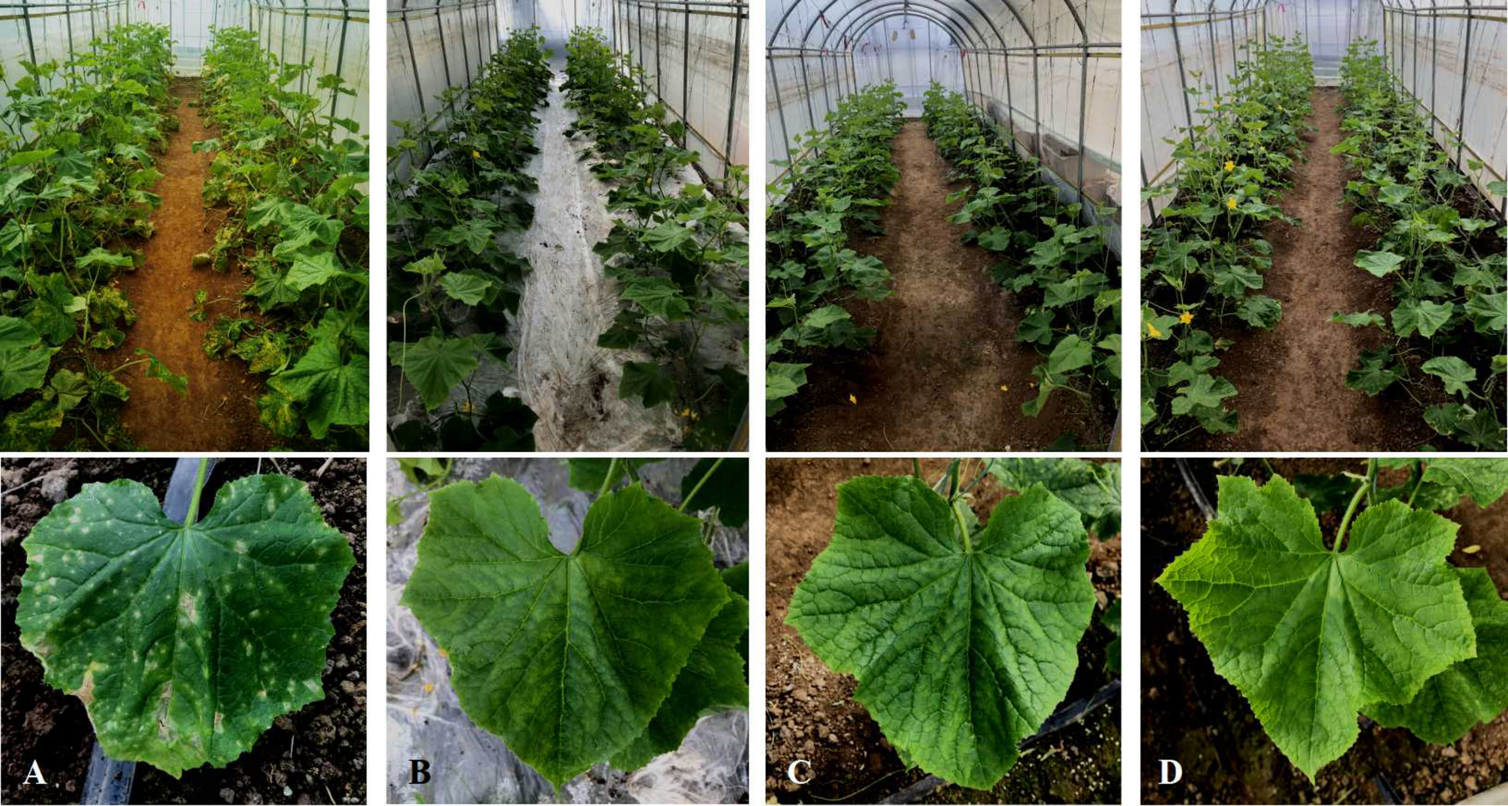
Effects of different soil treatments on leaf spot of cucumber seedlings. **(A)** Plant debris was mixed with sterile soil (CC). **(B)** Plant debris was mixed into the soil and covered with polyethylene film (CC-PE). **(C)** Plant debris was mixed into the soil, and CaCN_2_(CC-CaCN_2_) was added. **(D)** Natural soil without any treatment (CK).

We observed sporadic yellow spots at 35 DAT in the CC treatment, with a DI of 5.62. The DI gradually increased at 50 – 70 DAT, increasing from 22.44 at 50 DAT to 36.88 at 70 DAT. Cucumber leaves were covered with yellow spots, which is in line with the typical symptoms of cucumber Corynespora blight (**Figure 3**). Symptoms of disease appeared in the leaves in the CC-CaCN_2_ treatment at 50 DAT, with a DI of 1.13, and the DI was 2.46 at 70 DAT. In addition, there was no cucumber Corynespora blight in the CC-PE and CK treatments, which grew normally during the test (**Table 5**). These results indicate that CC-CaCN_2_ and CC-PE, especially CC-PE, could more effectively block the spread of *C. cassiicola* in soil to leaves and prevent cucumber disease.

**Table 5:** Effects of different soil treatments on the disease index (DI) of cucumber Corynespora blight.

Treatment ^a^	20 DAT ^b^	35 DAT	50 DAT	65 DAT	70 DAT
CC	0.00	5.62	22.44	25.15	36.88
CC-PE	0.00	0.00	0.00	0.00	0.00
CC-CaCN_2_	0.00	0.00	1.13	1.16	2.46
CK	0.00	0.00	0.00	0.00	0.00

^a^ Treatment: CC: plant debris was mixed with sterile soil. CC-PE: plant debris was mixed into the soil and covered with polyethylene film. CC-CaCN_2_: plant debris was mixed into the soil, and CaCN_2_ was added. CK: natural soil without any treatment.

^b^ DAT, day after treatment.

## 4 Discussion

Corynespora blight is an important disease that mainly infects cucumber leaves and spreads through air or seed-borne transmission (Yu, 1981; Wang et al., 2013). In fact, wind and rain have been noted as important agents of dispersal of plant pathogen spores (Aylor, 1990). Since the disease affects leaves, crop residues may also be a source of inoculum for the onset of Corynespora blight in the field. Previous research has reported that the disease debris of peanut scab disease (*Sphaceloma arachidis*) is added to the soil. An investigation found that the incidence rate of peanut scab was high and that the disease was serious approximately 150 days after planting (Kearney et al., 2002). After burying the debris of dahlia smut disease into the soil, 15% of healthy plants exhibited symptoms of the disease after 4–6 weeks (Okaisabor, 1969). At present, research on the disease of infected leaves transmitted by wind or rain in soil has been reported, including *Alternaria linicola*, *Cladosporium allii*, and *Colletotrichum acutatum* (Yoshiaki, 1994; Vloutoglou et al., 2010; Jordan et al., 2010). In this study, the plant debris of Corynespora blight was mixed with soil, and disease spots appeared during the growth of cucumber, with DI values of 48.27 and 36.88. The analysis of the combined results of the pot and greenhouse experiments indicated that soil residues are a source of inoculum for the onset and development of Corynespora blight. Subsequently, diseased cucumber plants can become secondary inoculum sources during the growing season. *C. cassiicola* was collected and detected in the air of the greenhouse where the disease residue soil was mixed in the greenhouse by the PMA method, which revealed that the pathogens in the soil could be transmitted to the air. Will the residues of gray mold, downy mildew, early blight or leaf spot of vegetables also spread and infect plant leaves? The significance of wind has been noted in the dispersal of pathogens from the inoculum source in splash droplets increasing and the size of the inoculum particles decreasing (Aylor, 1990). High greenhouse humidity causes guttation raindrops to form from plant leaves (Singh, 2016) or film droplets to shed, which may also cause the spread of diseased soil residues (Gilet and Bourouiba, 2015; Mukherjee et al., 2021). However, the distinction between rain splash or wind as the dispersal mechanism for spores is not always clear and needs to be further verified.

The suppressive effects of CaCN_2_ on soil-borne diseases have been observed in a variety of crops (Shi et al., 2009; Hu et al., 2021). However, its effect on soil debris has rarely been studied. Herein, we showed that CC-CaCN_2_ treatment could effectively inhibit the growth of Corynespora blight in soil from plant debris. The fertilizer CaCN_2_ can also be used to sanitize soils; thus, it has fungicidal activity (Shi et al., 2009). CaCN_2_ breaks down into hydrogen cyanamide in the soil, which is highly toxic to soil microbes (Shi et al., 2009). We observed that CaCN_2_ can effectively reduce the pathogens in the soil residues, but it cannot completely kill them, indicating that CaCN_2_ has the dual effect of inhibiting soil-borne pathogens and soil residues. It is worth noting that Corynespora blight disease was controlled at a relatively low level throughout the cucumber growth cycle. For the cucumber leaves treated with CaCN_2_ in the pot experiment, the phenomenon of drug damage may be due to the small culture space and the poor effect of soil drying. The DI of cucumber Corynespora blight was 0 in the polyethylene film (CC-PE) treatment, which indicated that it can effectively block the transmission of *C. cassiicola* in the soil through the air and prevent infection of the leaves. However, *C. cassiicola* was still present in the soil in the CC-PE treatment, and the isolation of PE film blocks the transmission of soil residues (Yao et al., 2016).

In addition, specific primers were designed to produce a 109 bp PCR product, which has strong specificity for the detection of *C. cassiicola*. We observed that the final concentration of PMA and the exposure time were 80 μmol·L^-1^ and 10 min, respectively. This is inconsistent with the detection of viable plant pathogens in several previous studies (Tian et al., 2016; Han et al., 2018; Chai et al., 2020b), which could be caused by different types of pathogens. The PMA-qPCR detection method of Corynespora blight of cucumber established for the first time in this study has the advantages of rapid, efficient and accurate quantification and provides technical support for disease monitoring and early warning.

## 5 Conclusions

**Figure 4:**
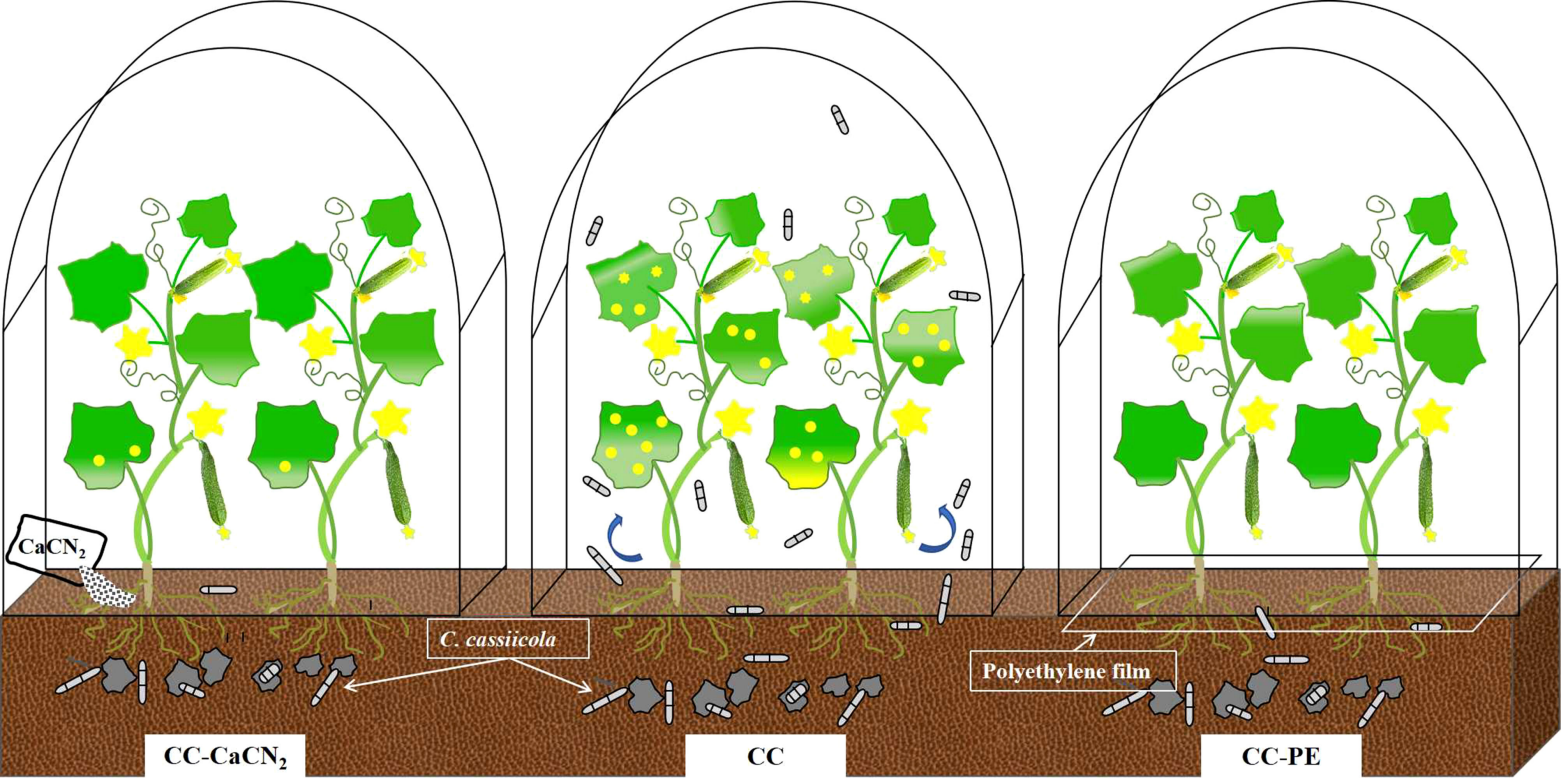
Schematic diagram of the retransmission and infection of plant leaf disease residues in soil.

In this study, we have revealed for the first time a novel mode of transmission, in which leaf blade disease residues in soil can spread again into the air and infect plants. Calcium cyanamide (CaCN_2_) and polyethylene film (PE) treatment, especially PE, could more effectively block the transmission of disease residues in soil to the air, resulting in less cucumber leaf disease (**Figure 4**). Considering the tremendous production scale of protected vegetable cultivation in China, reasonable technology for returning plant residues to the field would have wide application prospects.

## Data availability statement

The original contributions presented in the study are included in the article/**Supplementary Material**. Further inquiries can be directed to the corresponding author.

## Author contributions

XX and BL designed the experiments. LC and LL conducted the field and molecular experiments. YS, AC, and TF analyzed the data. XX and LL prepared the plant materials used in this study. XX, LC, LL and BL wrote the manuscript. All authors contributed to the article and approved the submitted version.
