# Supplementary material for: The calcium cyanamide and polyethylene blocks the secondary transmission and infection of vegetable leaf diseases
Source: Front Plant Sci. 2022 Dec 20;13:1027584. doi: 10.3389/fpls.2022.1027584 (PMC9807914; doi:10.3389/fpls.2022.1027584)
Supplement: Supplementary file 1 [file Table_1.docx]

**Table S1** Fungal strains for the PCR specificity test.

| Species | Isolate Code ^a^ | Host | Geographic origin | Ct |
| --- | --- | --- | --- | --- |
| *Corynespora cassiicola* | HG2010061201 | *Cucumis sativus* | Shandong, China | 18.37 |
| *Pseudoperonospora cubensis* | HG2020040705 | *Cucumis sativus* | Beijing, China | >35.00 |
| *Sphaerotheca cucurbitae* | HG2014100902 | *Cucumis sativus* | Beijing, China | >35.00 |
| *Pseudomonas syringae* | HG2018051101 | *Cucumis sativus* | Hebei, China | >35.00 |
| *Fusarium oxysporum* | HG2018060701 | *Cucumis sativus* | Tianjin, China | >35.00 |
| *Alternaria cucumerina* | HG2019081101 | *Cucumis sativus* | Shandong, China | >35.00 |
| *Cladosporium cucumerinum* | HG2019090701 | *Cucumis sativus* | Hainan, China | >35.00 |
| *Colletotrichum orbiculare* | HG2010061201 | *Cucumis sativus* | Beijing, China | >35.00 |

^a^ type strain
